# Suppression of experimental cerebral malaria by disruption of malate:quinone oxidoreductase

**DOI:** 10.1186/s12936-017-1898-5

**Published:** 2017-06-12

**Authors:** Mamoru Niikura, Keisuke Komatsuya, Shin-Ichi Inoue, Risa Matsuda, Hiroko Asahi, Daniel Ken Inaoka, Kiyoshi Kita, Fumie Kobayashi

**Affiliations:** 1 Department of Infectious Diseases, Kyorin University School of Medicine, 6-20-2 Shinkawa, Mitaka, Tokyo 181-8611 Japan; 2 School of Tropical Medicine and Global Health, Nagasaki University, Nagasaki, 852-8523 Japan; 3 Department of Biomedical Chemistry, The University of Tokyo, Tokyo, 113-0033 Japan

**Keywords:** *Plasmodium berghei*, Fumarate hydratase (FH), Malate:quinone oxidoreductase (MQO), Luciferase–luciferin system

## Abstract

**Background:**

Aspartate, which is converted from oxaloacetate (OAA) by aspartate aminotransferase, is considered an important precursor for purine salvage and pyrimidine de novo biosynthesis, and is thus indispensable for the growth of *Plasmodium* parasites at the asexual blood stages. OAA can be produced in malaria parasites via two routes: (i) from phosphoenolpyruvate (PEP) by phosphoenolpyruvate carboxylase (PEPC) in the cytosol, or (ii) from fumarate by consecutive reactions catalyzed by fumarate hydratase (FH) and malate:quinone oxidoreductase (MQO) in the mitochondria of malaria parasites. Although PEPC-deficient *Plasmodium falciparum* and *Plasmodium berghei* (rodent malaria) parasites show a growth defect, the mutant *P. berghei* can still cause experimental cerebral malaria (ECM) with similar dynamics to wild-type parasites. In contrast, the importance of FH and MQO for parasite viability, growth and virulence is not fully understood because no FH- and MQO-deficient *P. falciparum* has been established. In this study, the role of FH and MQO in the pathogenicity of asexual-blood-stage *Plasmodium* parasites causing cerebral malaria was examined.

**Results:**

First, FH- and MQO-deficient parasites were generated by inserting a luciferase-expressing cassette into the *fh* and *mqo* loci in the genome of *P. berghei* ANKA strain. Second, the viability of FH-deficient and MQO-deficient parasites that express luciferase was determined by measuring luciferase activity, and the effect of FH or MQO deficiency on the development of ECM was examined. While the viability of FH-deficient *P. berghei* was comparable to that of control parasites, MQO-deficient parasites exhibited considerably reduced viability. FH activity derived from erythrocytes was also detected. This result and the absence of phenotype in FH-deficient *P. berghei* parasites suggest that fumarate can be metabolized to malate by host or parasite FH in *P. berghei*-infected erythrocytes. Furthermore, although the growth of FH- and MQO-deficient parasites was impaired, the development of ECM was suppressed only in mice infected with MQO-deficient parasites.

**Conclusions:**

These findings suggest that MQO-mediated mitochondrial functions are required for development of ECM of asexual-blood-stage *Plasmodium* parasites.

**Electronic supplementary material:**

The online version of this article (doi:10.1186/s12936-017-1898-5) contains supplementary material, which is available to authorized users.

## Background

*Plasmodium* species are among the most important mosquito-borne pathogens worldwide, and cause an estimated 212 million malaria cases and 429,000 deaths due to malaria per year [1]. When an infected mosquito takes a blood meal, a small number of *Plasmodium* sporozoites are injected into the host’s bloodstream. The injected sporozoites invade hepatocytes and produce merozoites. These merozoites are released into the bloodstream and invade erythrocytes, in which the vast majority multiply asexually; only a small subset of parasites differentiate into sexual precursor cells (i.e., male and female gametocytes [2]).

The metabolic pathways in *Plasmodium* parasites differ from those of their host. These parasites use nutrients obtained from the host [3] and, to sustain parasite growth, adenosine triphosphate (ATP) is produced (mainly by glycolysis). Although *Plasmodium* spp. possesses all of the genes necessary for the tricarboxylic acid (TCA) cycle, [4] and most of the genes needed for electron transport chain (ETC) enzymes, asexual-blood-stage malaria parasites rely mainly on cytosolic glycolysis with limited contribution from mitochondrial oxidative phosphorylation for ATP synthesis [5, 6]. Several reports [7–9] have demonstrated that the TCA cycle is not essential for survival of asexual-blood-stage parasites, but is required for survival of mosquito-stage parasites. However, two of the eight mitochondrial TCA cycle enzymes, fumarate hydratase (FH) and malate:quinone oxidoreductase (MQO), could not be genetically ablated in asexual-blood-stage *Plasmodium falciparum*, suggesting that these two enzymes are promising drug targets [9].

In this regard, the “fumarate cycle” should be noted (see Additional file 1). The purine salvage pathway is an important source of fumarate, which is generated as a by-product of the adenylosuccinate lyase reaction in *Plasmodium* [10] (see Additional file 1). This fumarate can then be converted into malate by the malarial FH [11], and then to OAA by MQO in mitochondria [12] (see Additional file 1). OAA generated by MQO is converted to aspartate by aspartate aminotransferase (AAT) in the cytosol, which feeds the purine salvage pathway, through which fumarate is regenerated, in a process termed the fumarate cycle [11] (see Additional file 1). The oxidation of malate to oxaloacetate by MQO is coupled to reduction of ubiquinone (UQ) to form ubiquinol (UQH_2_), which then feeds into the ETC at complex III [11, 12]. This purine salvage pathway, TCA cycle and ETC network suggests the presence of intense metabolic cross-talk in *Plasmodium* parasites [11].

OAA can be produced in malaria parasites from (i) fumarate by consecutive reactions catalyzed by FH and MQO in the mitochondria of malaria parasites, as described above, or from (ii) phosphoenolpyruvate (PEP; common in plants and bacteria) by phosphoenolpyruvate carboxylase (PEPC) in the cytosol of the parasite. PEPC-deficient *P. falciparum* has a severe growth defect. In contrast, growth of PEPC-deficient *P. falciparum* is partially rescued by supplementation of cultures with a high concentration of fumarate or malate [13]. Interestingly, PEPC-deficient *Plasmodium berghei* cause severe cerebral malaria, with dynamics similar to those caused by wild-type parasites [14]. However, the importance of FH and MQO for asexual-stage parasite viability and growth in cerebral malaria is unclear.

In this study, FH and MQO in *P. berghei* (strain ANKA), which is the aetiologic agent of experimental cerebral malaria (ECM) in rodents were focused. To investigate the roles of FH and MQO in the viability and growth of malaria parasites, a luciferase-expressing cassette was introduced into the *mqo* and *fh* loci in the genome of *P. berghei*, and the viability of *fh*-disrupted (Δ*fh*) and *mqo*-disrupted (Δ*mqo*) parasites was evaluated by measuring luciferase activities. Moreover, the effect of FH and MQO deficiency on the development of ECM caused by *P. berghei* were assessed.

## Methods

### Mice

Female C57BL/6J mice 5- to 6-weeks old were purchased from CLEA Japan INC (Tokyo, Japan). The experiments were approved by the Experimental Animal Ethics Committee of Kyorin University School of Medicine, Tokyo, and all experimental animals were kept at the animal facility in a specific-pathogen-free unit with sterile bedding, food and water.

### DNA constructs

The SK-1 construct contained a selection cassette consisting of the green fluorescent protein gene (*gfp*) and a pyrimethamine resistance gene, human dihydrofolate reductase-thymidylate synthase (*hdhfr*) [15]. The expression of *gfp* and *hdhfr* is controlled by *hsp70* (PBANKA_071190) and *elongation factor*-*1* (PBANKA_113340) promoters, respectively. Plasmid (pLG4.10[*luc2*]) containing luciferase gene (*luc2*) was purchased from Promega (Madison, WI, USA). To generate luciferase-expressing cassette, *luc2* was amplified by PCR using specific primers (see Additional files 2, 3). The PCR product of *luc2* and SK-1 construct were cleaved using the NheI and BglII restriction enzymes, and the *gfp* of SK-1 was replaced with *luc2*. The gene-targeting vectors were prepared by PCR [16]. Briefly, the 5′ and 3′ flanking regions the ORF of the target genes, *p230* [17], *fh* locus (PBANKA_082810) and *mqo* locus (PBANKA_111630), were amplified by PCR. The PCR products were annealed to either side of luciferase expressing cassette and amplified by PCR using gene-specific primers (see Additional file 2).

### Parasites and infections

*Plasmodium berghei* ANKA strain is a high-virulence strain and the parasites, which had been cloned by limiting dilution, were obtained from Dr. W. P. Weidanz (University of Wisconsin–Madison, Madison, WI, USA). Erythrocytes infected with *P. berghei* parasites were transferred to RPMI1640 medium supplemented with 25% (v/v) FBS, 0.05 mg/mL Penicillin, 0.05 mg/mL Streptomycin and then incubated for 18 or 22 h under condition of 90% N_2_, 5% CO_2_ and 5% O_2_. Mature schizonts and gametocytes were collected by Nycodenz density-gradient centrifugation [18]. Transformations were performed using the Amaxa Basic Parasite Nucleofector Kit (Amaxa GmbH, Cologne, Germany) according to the manufacturer’s protocol and luciferase-expressing cassette was introduced into the ORF of targeted gene by double-crossover homologous recombination [18]. Briefly, 5 × 10^6^ to 5 × 10^7^ purified *P. berghei* mature schizonts were mixed with 100 μL of Nucleofector solution containing 5 μg of a gene-targeting vector. Transfections were then completed using the Amaxa Nucleofector electroporation program U-33. Transfected parasites were then injected intravenously (i.v.) into naïve C57BL/6 recipient mice. At 30 h post-injection, transfected parasites were selected by addition of pyrimethamine to the drinking water of infected mice. After parasitaemia returned to detectable levels post-selection, transfected parasites were cloned by limiting dilution, after which a single parasite was injected into a mouse to ensure a clonally pure population. Cloned transfected parasites were stored as frozen stocks in liquid nitrogen. Erythrocytes infected with transfected parasites were generated in donor mice inoculated intraperitoneally with each frozen stock of parasites. The donor mice were monitored for parasitaemia daily and bled for experimental infection in ascending periods of parasitaemia. Experimental mice were infected intravenously with 1 × 10^4^ infected erythrocytes or 5 × 10^6^ to 5 × 10^7^ purified mature schizonts of a given parasite strain.

### Parasitaemia

Blood was observed by microscopic examination of methanol-fixed tail blood smears stained with 3% (w/v) Giemsa diluted with Sörensen’s phosphate buffer, pH 7.2, for 45 min. The number of infected erythrocytes in 250 erythrocytes was enumerated when parasitaemia exceeded 10%, whereas 1 × 10^4^ erythrocytes were examined when mice showed lower parasitaemia. The percentage of parasitaemia was calculated as follows: [(Number of infected erythrocytes)/(Total number of erythrocytes)] × 100.

### Genomic PCR

To generate luciferase-expressing cassette and confirm the introduction of Luc2-expressing cassette into target gene, genomic PCR was performed using a 25 μL PCR mixture containing 1×TaKaRa PrimeSTAR GXL buffer (TaKaRa, Shiga, Japan), 2.5 mM dNTPs, 0.5 μL of DNA, 5 U/μL TaKaRa PrimeSTAR GXL DNA polymerase (TaKaRa), and PCR primers (0.25 μM). Thirty-five cycles of PCR were performed on a C1000 thermal cycler (Bio-Rad, Hercules, CA, USA). Each cycle consisted of denaturation at 98 °C for 15 s, annealing at 55 °C for 15 s, and extension at 68 °C for 1–6 min. PCR products were then analysed on a 1% (w/v) agarose gel, and stained with ethidium bromide.

### Semi-quantitative RT-PCR

Blood was obtained from infected mice exhibiting 2–5% parasitaemia. Total RNA was isolated from blood containing 5 × 10^6^ or 1 × 10^7^ infected erythrocytes using Isogen (Nippon Gene, Tokyo, Japan) according to the manufacturer’s protocol. Total RNA was then treated with DNase and reverse-transcribed using murine leukaemia virus reverse transcriptase (Applied Biosystems, Carlsbad, CA, USA) with random hexamer primers under the following conditions: 70 °C for 10 min, 25 °C for 10 min, and 42 °C for 30 min. The reaction was terminated by heating at 99 °C for 5 min, and the resulting cDNA products were stored at −20 °C until required. All PCR reactions were run in a 25 μL volume consisting of 1×TaKaRa Ex Taq buffer, 2.5 mM dNTP, 1 μL of cDNA products, 5 U/μL TaKaRa Ex Taq DNA polymerase, and PCR primers (0.25 μM); a list of primers used for semi-quantitative RT-PCR can be found in Additional file 2. PCR reactions were performed on a C1000 thermal cycler (Bio Rad) for 30 cycles under the following conditions: denaturation at 95 °C for 30 s, annealing at 55 °C for 30 s and extension at 72 °C for 45 s. Products were resolved on a 2% (w/v) agarose gel, and stained with ethidium bromide. Samples with DNase-treated RNA template were used as the negative control.

### Preparation of mitochondria

To remove leukocytes, the blood was mixed with same volume of PBS and passed over a Plasmodipur Filter (EuroProxima, Arnhem, Netherlands). Erythrocytes were washed twice with RPMI1640 medium by centrifugation at 4 °C at 800×*g*, for 5 min and then transferred to RPMI1640 medium supplemented with 25% (v/v) FBS, 0.05 mg/mL Penicillin, 0.05 mg/mL Streptomycin. Then erythrocytes were incubated for 16 h under condition of 90% N_2_, 5% CO_2_ and 5% O_2_. Stages of parasite were mainly schizonts as confirmed by Giemsa staining. Infected parasites were collected by centrifugation at 4 °C at 800×*g*, for 5 min. Crude mitochondria of *P. berghei* were prepared as described previously [19].

### Measurement of MQO activity

MQO activity assay was performed at 25 °C with V-660 spectrophotometer (JASCO) and measured in 1 mL of the reaction mixture containing 20 µg of mitochondrial fraction, 45 µM 2,6-dichlorophenolindophenol (DCIP), 100 µM ubiquinone-2 and 2 mM KCN in 50 mM potassium phosphate buffer, pH 8.0 by recording the decrease in absorbance due to DCIP reduction at 600 nm (ε600 = 21/mM/cm) after the reaction was initiated by adding 10 mM sodium malate.

### Measurement of FH activity

FH activity was performed 37 °C with Benchmark Plus microplate spectrophotometer (Bio-Rad) and measured in 200 µL of the reaction mixture containing 20 µg of mitochondrial fraction, 0.25% (v/v) Triton X-100, 50 µg/mL 3-acetylpyridine-adenine dinucleotide, oxidized form (APAD^+^) (Oriental Yeast, Japan), 1 U/mL diaphorase, 0.2 mg/mL nitroblue tetrazolium (NBT) and 2 U/mL malate dehydrogenase from pig heart (Oriental Yeast) in 100 mM Tris–HCl buffer pH 8.0 by recording the absorbance change of NBT at 530 nm (ε530 = 3.6 × 10^4^/mM/cm) after the reaction was initiated by adding 10 mM sodium fumarate.

### Luciferase assay for evaluation of the viability of malaria parasites

To monitor the viability of *fh*- and *mqo*-disrupted *P. berghei* by luciferase activities, luciferase-expressing cassette was introduced into *fh* locus (PBANKA_082810) and *mqo* locus (PBANKA_111630) in the genome of *P. berghei* (see Additional file 3). Moreover, luciferase-expressing cassette was also introduced into *p230* locus (PBANKA_030600), which is not an essential gene in the complete life cycle of *P. berghei* [17], and the resultant was used as the control parasite in the following experiments. Blood was obtained from C57BL/6 mice exhibiting 2–5% parasitaemia, and diluted with RPMI 1640 medium to a final concentration of 1 × 10^7^ infected erythrocytes/well in a 96-well plate. Then, infected erythrocytes were cultured for 3 h in RPMI 1640 medium supplemented with 5 mM sodium fumarate (Sigma-Aldrich Co, Missouri, USA), 5 mM sodium malate (Sigma-Aldrich Co) or control PBS. After cultivation, d-luciferin (1.5 mM; Promega) was added to each well, and luminescence measured using an SH9000 luminometer (Hitachi High-Technologies Corporation, Tokyo, Japan) at 5 min after addition of d-luciferin. Results are presented as relative fluorescence unit (RFU) fold change compared with control PBS at 5 min after addition of d-luciferin. Also, blood was obtained from C57BL/6 mice on 16 h after injection of schizonts, and diluted with RPMI 1640 medium to a final concentration of 5 × 10^6^ infected erythrocytes/well in a 96-well plate. After cultivation, d-luciferin (1.5 mM; Promega) was added to each well, and luminescence measured using an SH9000 luminometer at 20 min after addition of d-luciferin. Results are presented as absolute luciferase activity in counts per second (cps).

### Determination of mitochondrial membrane potential

To investigate whether mitochondrial membrane potential (MMP) in malaria parasites is compromised by deficiency of FH or MQO, MMP in trophozoites of control, Δ*fh* and Δ*mqo* parasites was determined using MMP-sensitive fluorochrome Mitotracker^®^ Red CMXRos (Invitrogen, Darmstadt, Germany). Inner membrane and nuclear DNA were then stained with the BODIPY FL C16 (Invitrogen) and Hoechst 33342 (Invitrogen), respectively. Mitotracker^®^ Red CMXRos was added to medium with a concentration of 100 nM and incubated for 30 min at 37 °C. Then, BODIPY FL C16 and Hoechst 33342 were added to medium with a concentration of 100 nM and of 1 µg/mL for 10 min at 37 °C. For all staining of malaria parasites, the staining medium was removed after the incubation period and the fresh medium was added. The Bright field and fluorescence microscopy images were photographed at 1000× magnification using an All-in-One Fluorescence Microscope (BZ9000; KEYENCE Japan, Osaka, Japan). Mean fluorescence intensity (MFI) of MitoTracker in the photographs was analysed using BZ-II Analyzer software (KEYENCE Japan).

### Examination of the blood–brain barrier

It is known that breakdown of the blood–brain barrier is an indicator of ECM. When *P. berghei*-infected mice develop ECM, Evans blue is injected i.v. and the brain is stained as a result of extravasation of the dye [20]. Mice were injected i.v. with 0.2 mL of 1% (w/v) Evans blue (Wako, Osaka, Japan) on days 7 and 14 post-infection. Mice were euthanized and brains perfused with PBS 1 h later. After the brains were removed and photographed they were weighed and placed in 2 mL formamide (Wako, Osaka, Japan) at 37 °C for 48 h to extract the Evans blue dye. Absorbance was measured at λ = 620 nm with a Multiscan FC microplate reader (Thermo Fisher Scientific Inc., Waltham, USA). The Evans blue concentration was calculated from a standard curve and is expressed as µg of Evans blue per g of brain.

### Statistical analysis

Student’s t test was performed using Statcel (OMS Ltd., Saitama, Japan). Statistically significant differences were defined as a value of p < 0.05.

## Results

### Generation of FH-disrupted and MQO-disrupted *Plasmodium berghei*

**Fig. 1:**
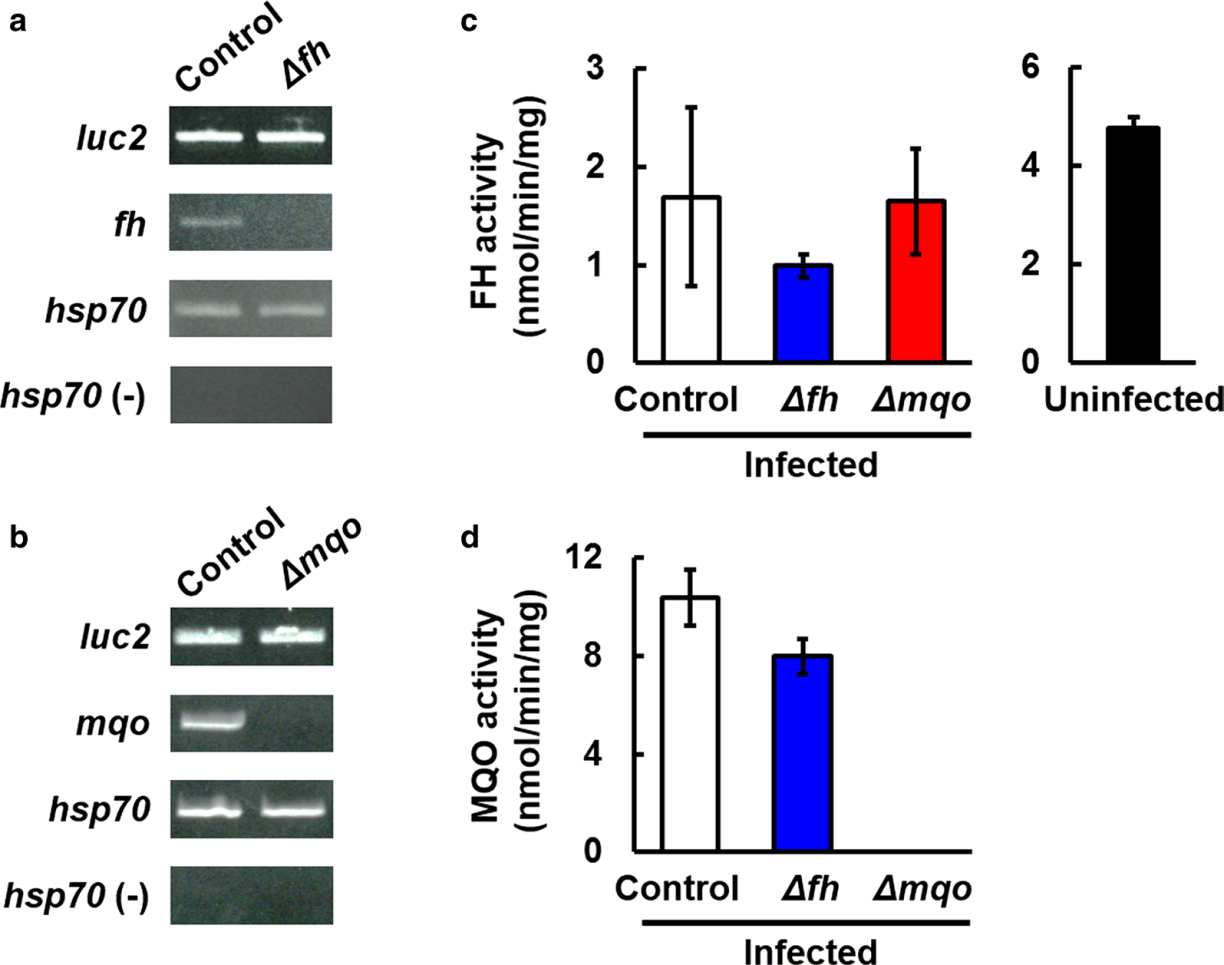
Establishment of fumarate hydratase (FH)- and malate:quinone oxidoreductase (MQO)-deficient *P. berghei*. **a** Expression of *luciferase* (*Luc2*) and *fh* in control and Δ*fh* parasites. **b** Expression of *Luc2* and *mqo* in control and Δ*mqo* parasites. The genes were subjected to semi-quantitative reverse-transcription polymerase chain reaction (RT-PCR) using specific primers (see Additional files 2, 3). *hsp70* was used as a positive control. Samples treated with DNase-treated RNA template [*hsp70* (−)] were used as a negative control that is the control of eventual DNA contamination of the RNA preparations. **c** FH activity in infected (*left*) and uninfected (*right*) erythrocytes. **d** MQO activity in infected erythrocytes. Results are expressed as mean ± SD of triplicate determinations

To investigate the role of FH and MQO in malaria parasite growth in erythrocytes, *fh*- and *mqo*-disrupted *P. berghei* were generated. The luciferase mRNA level in *fh*-disrupted (Δ*fh*) and *mqo*-disrupted (Δ*mqo*) parasites was comparable to that in control parasites (Fig. 1a, b). Moreover, *fh* and *mqo* mRNAs were not detected in Δ*fh* and Δ*mqo* parasites, respectively (Fig. 1a, b). These findings demonstrate that the luciferase-expressing cassette was successfully introduced into *fh* and *mqo* locus.

In Δ*fh* parasite-infected erythrocytes, the specific activity of FH was 0.99 ± 0.12 nmol/min/mg, which was 58.5 and 60.0% lower compared to control-infected and Δ*mqo* parasite-infected erythrocytes (1.69 ± 0.91 and 1.65 ± 0.54 nmol/min/mg, respectively) (Fig. 1c). As FH activity was detected in uninfected erythrocytes, the FH activity in Δ*fh* parasite-infected erythrocytes seems to be derived from host FH (Fig. 1c). The MQO activity in Δ*mqo* parasite-infected erythrocytes was completely abrogated, although high activities in control parasite- and Δ*fh* parasite-infected erythrocytes were detected (Fig. 1d).

### Inhibition of parasite growth during late trophozoites and schizont stage by deficiency of FH and MQO

**Fig. 2:**
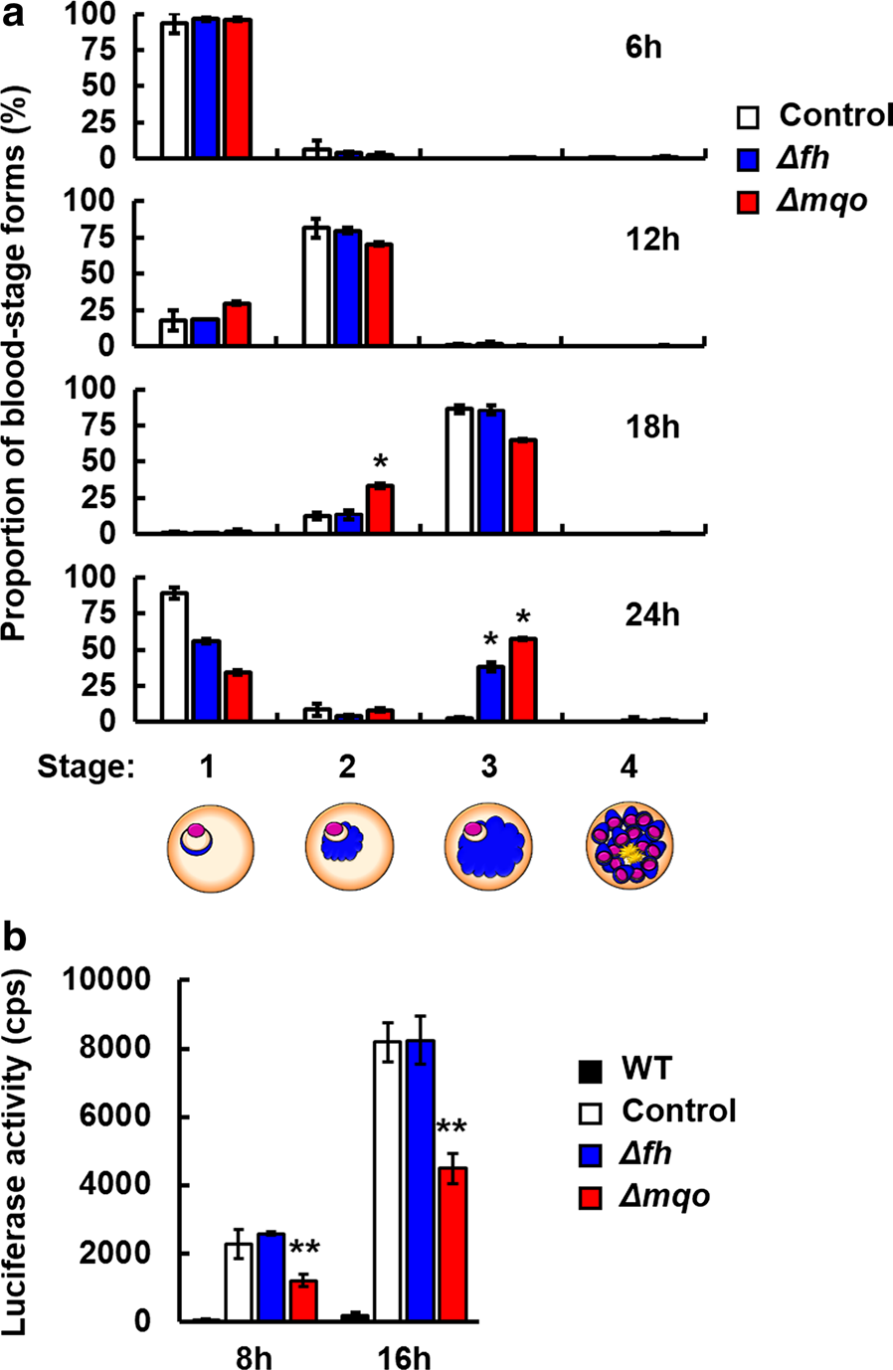
Parasite growth is delayed by deficiency of FH and MQO. **a** C57BL/6 mice were infected with 5 × 10^6^ to 5 × 10^7^ schizonts of control, Δ*fh* and Δ*mqo* parasites. At 6, 12, 18 and 24 h after inoculation, the proportion of asexual-blood-stage forms in 100 infected erythrocytes was determined. Results are expressed as mean ± SD of three mice. *Asterisks* indicate statistically significant differences (*, vs. control parasites). **b** Absolute luciferase activity values in trophozoites of wild-type (WT), control, Δ*fh* and Δ*mqo* parasites. Trophozoites (5 × 10^6^) were obtained from C57BL/6 mice at 8 and 16 h post-inoculation with purified schizonts. Results are expressed as mean ± SD absolute luciferase activity values of three wells at 20 min after addition of *d*-luciferin. Experiments were performed in duplicate, and representative data are shown. *Asterisks* indicate statistically significant differences (**, vs. control and Δ*fh* parasites)

The effect of deficiency of FH and MQO on parasite growth in vivo were investigated (Fig. 2a). Δ*fh* and Δ*mqo* parasites showed a growth pattern similar to control parasites, until 18 h post-inoculation of schizonts (Fig. 2a). However, at 24 h post-inoculation, the proportions of late trophozoites (stage 3) of Δ*fh* and Δ*mqo* parasites were considerably higher than in control parasites (Fig. 2a). These findings suggest that parasite growth during late trophozoites and schizont stage is delayed by deficiency of FH or MQO.

To evaluate the production of gametocytes in Δ*fh* and Δ*mqo* parasites, the mRNA levels of the gametocyte-specific proteins MDV1/PEG3 and g377 were examined. *mdv*-*1/peg3* and *g377* mRNAs can be detected from the early phase of gametocytogenesis [21, 22]. As results, gametocyte-specific gene expression during asexual-blood-stage Δ*fh* and Δ*mqo* parasites was comparable to that of control parasites (see Additional file 4A, B). Nuclear enlargement, the distribution of pigment granules throughout the cytoplasm and enlargement of cells were observed in a 22 h culture of Δ*fh* and Δ*mqo* parasites (see Additional file 4C, D). These three features are gametocyte-specific characteristics [23]. Taken together, these results suggest that the development of female and male gametocytes of malaria parasites was not affected by deficiency of FH and MQO.

### Effect of FH and MQO deficiency on the viability of malaria parasites

ATP plays a central role in energy transduction in both eukaryotic and prokaryotic cells, and is produced in all metabolically active cells. The luciferase–luciferin system, in which luciferase reacts with *d*-luciferin in the presence of ATP, an oxygen molecule, and magnesium ions to produce luminescence, has facilitated investigation of parasite viability [24]. To examine the effect of deficiency of FH and MQO on parasite viability, schizonts of control, Δ*fh* and Δ*mqo* parasites were inoculated into mice. Trophozoites were obtained at 8 and 16 h post-inoculation and their luciferase activities were measured. The absolute luciferase activity values of Δ*fh* parasites were comparable to those of control parasites (Fig. 2b). In contrast, the luciferase activities of Δ*mqo* parasites were significantly reduced, by ~50% compared with those of control and Δ*fh* parasites (Fig. 2b). These findings suggest that the viability of asexual-blood-stage malaria parasites is decreased by deficiency of MQO but not FH.

**Fig. 3:**
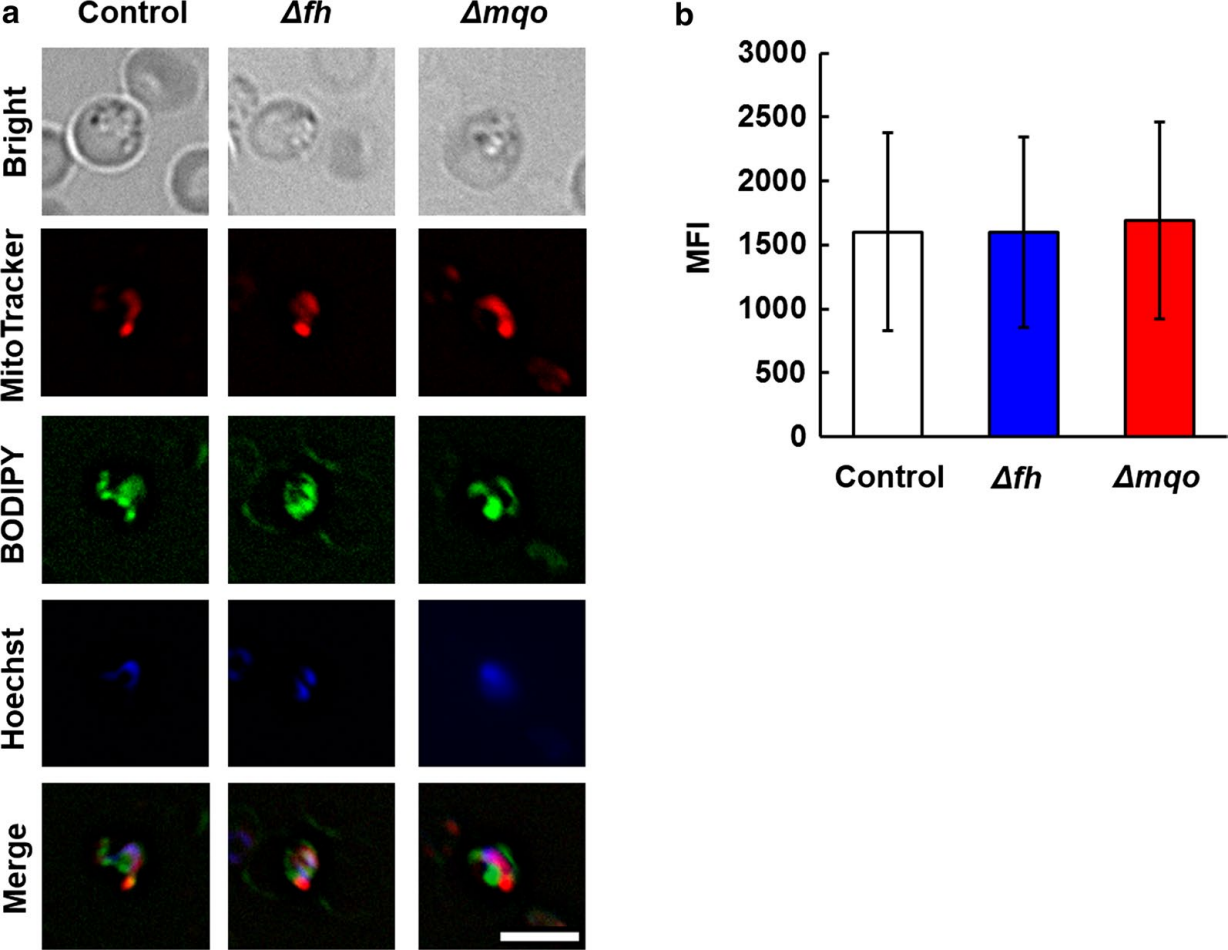
Mitochondrial membrane potential (MMP) in trophozoites of control, Δ*fh* and Δ*mqo* parasites. **a** Bright-field and fluorescence microscopy images of control, Δ*fh* and Δ*mqo* parasites (*left panel*). MMP was assessed using the MMP-sensitive fluorochrome MitoTracker^®^ Red CMXRos (MitoTracker, *red*). The inner membrane and cell nuclei were stained with BODIPY FL C16 (BODIPY, *green*) and Hoechst 33342 (Hoechst, *blue*), respectively. *Bar* indicates 5 µm. **b** Mean fluorescence intensity (MFI) of MitoTracker (*right panel*). Results are expressed as mean ± SD of 100 control, Δ*fh* and Δ*mqo* trophozoites. Experiments were performed in duplicate, and representative data are shown

To investigate the effect of FH and MQO deficiency on mitochondrial function, the study compared the mitochondrial membrane potential (MMP) of control, Δ*fh* and Δ*mqo* parasites by monitoring MitoTracker uptake. MMP was not affected by deficiency of FH and MQO (Fig. 3), suggesting that the reduction of parasite viability caused by MQO deficiency is independent of defects in mitochondrial integrity.

### Effect of fumarate and malate on the viability of control, Δ*fh* and Δ*mqo* parasites

**Fig. 4:**
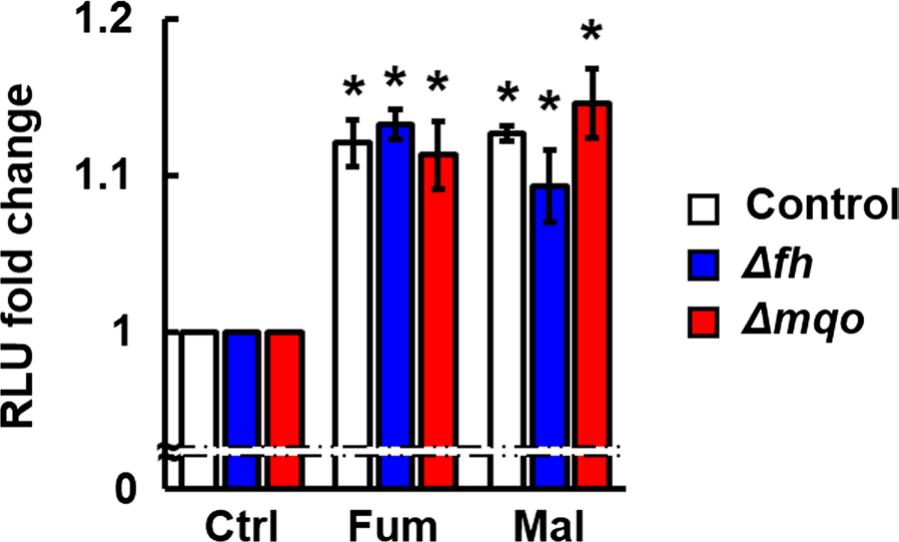
Parasite viability is restored by addition of fumarate or malate. Fold change of luciferase activity in control, Δ*fh* and Δ*mqo* parasites after cultivation. Infected erythrocytes (1 × 10^7^) were cultured for 3 h in RPMI 1640 medium supplemented with 5 mM fumarate (Fum), 5 mM malate (Mal) or control PBS (Ctrl). Results are expressed as mean ± SD fold changes of three wells in relative luciferase units (RLU) compared with the control at 5 min after addition of *d*-luciferin. Experiments were performed in duplicate, and representative data are shown. *Asterisks* indicate statistically significant differences (*, vs. control)

Results that luciferase activities were not affected by deficiency of FH suggest cooperation of the host- and parasite-derived FH in fumarate metabolism. Therefore, the effect of addition of fumarate or malate to the culture on the viability of control, Δ*fh* and Δ*mqo* parasites were investigated using the luciferase–luciferin system. In the culture of control parasites, luciferase activities were increased by addition of fumarate or malate compared with the control (Fig. 4). The luciferase activities of Δ*fh* parasites and Δ*mqo* parasites were also increased in culture medium supplemented with fumarate or malate (Fig. 4). Therefore, enriched metabolic substitutions by host-derived enzymes, such as FH, in erythrocytes may be associated with increased viability of Δ*fh* and Δ*mqo* parasites in culture medium supplemented with fumarate or malate.

### Suppression of ECM development by deficiency of MQO but not FH

**Fig. 5:**
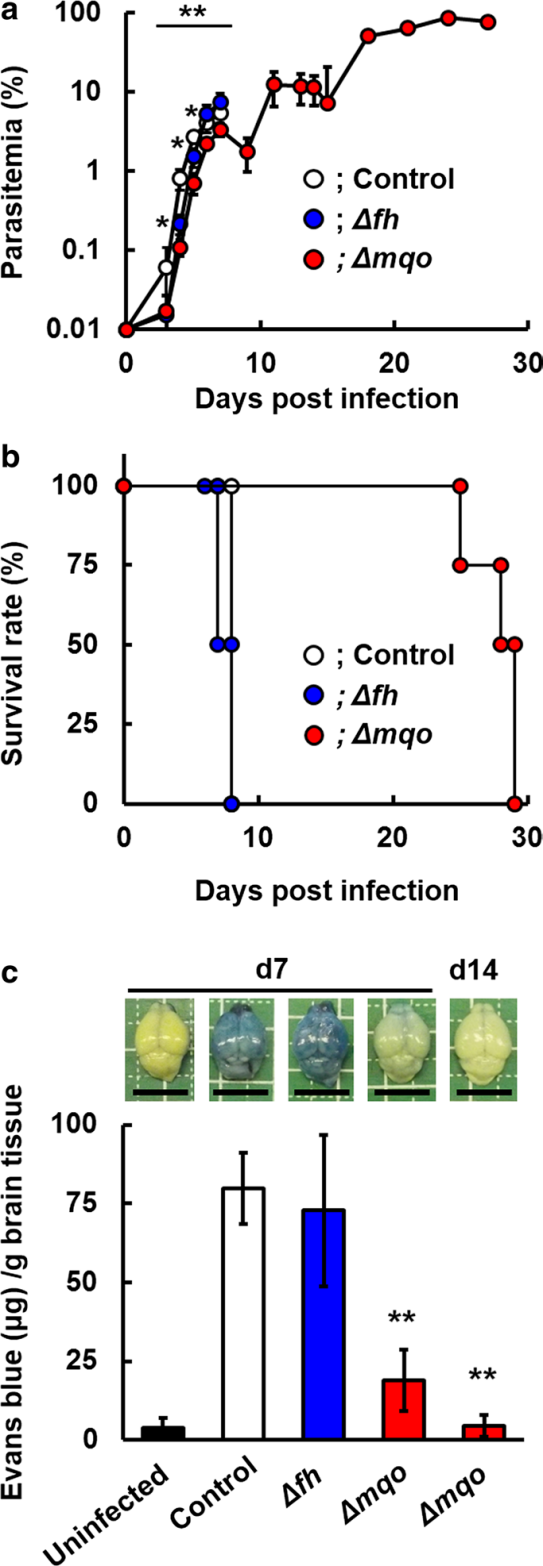
Experimental cerebral malaria (ECM) is suppressed by deficiency of parasite MQO but not FH. Female C57BL/6 mice were infected with 1 × 10^4^ infected erythrocytes of control, Δ*fh* and Δ*mqo* parasites. **a** Course of parasitaemia. *Asterisks* indicate a statistically significant difference (*, control vs. Δ*fh*-infected mice; **, control vs. Δ*mqo*-infected mice). **b** Survival rate. Note that neurologic signs of ECM were observed in mice infected with control and Δ*fh* parasites on day 7 post-inoculation. **c** Brains of mice injected with Evans blue (*top panels*). Brains were obtained from uninfected and control- and Δ*fh*-infected mice on day 7 post-infection, and from Δ*mqo*-infected mice on days 7 and 14 post-infection. *Scale bar* 10 mm. Quantitative analysis of Evans blue extravasation in the brain (*bottom*). Results are expressed as mean ± SD of three mice. Experiments were performed in duplicate, and representative data are shown. *Asterisks* indicate statistically significant differences (**, control vs. Δ*mqo*-infected mice)

*Plasmodium berghei* strain ANKA is the causative agent of ECM, in which neurologic signs and histopathological findings, such as haemorrhages and sequestration of infected erythrocytes within cerebral microvessels, are observed [20]. However, the relationship between parasite growth and pathogenicity in ECM is unclear. Therefore, the effect of FH or MQO deficiency on ECM were examined. Mice infected with Δ*fh* and Δ*mqo* parasites showed lower levels of parasitaemia than mice infected with control parasites until days 5 and 7 post-infection, respectively (Fig. 5a; see Additional file 5). All mice infected with Δ*fh* parasites showed neurologic signs and died on days 6–7 post-infection (Fig. 5b); this is similar to the effect in control mice. In contrast, the survival of mice infected with Δ*mqo* parasites was prolonged compared to that of mice infected with control parasites or Δ*fh* parasites (Fig. 5b). The mice infected with Δ*mqo* parasites ultimately died via anaemia within days 25–30 post-infection.

Breakdown of the blood–brain barrier is an indicator of ECM [20]; therefore, breakdown of the blood–brain barrier in mice infected with control, Δ*fh* or Δ*mqo* parasites were investigated by assessing extravasation of Evans blue in the brain. The levels of extravasation of Evans blue in the brains of Δ*fh*-infected mice on day 7 post-infection were comparable to those in mice infected with control parasites (Fig. 5c). In contrast, the levels of extravasation of Evans blue in the brains of Δ*mqo*-infected mice on days 7 and 14 post-infection were markedly lower than those in mice infected with control or Δ*fh* parasites, and were comparable to those of uninfected mice (Fig. 5c). These results suggest that the ECM caused by malaria parasites is suppressed by deficiency of parasite MQO, but not FH.

## Discussion

This study demonstrated that the growth of *P. berghei* was delayed by deficiency of FH and MQO, and that the development of ECM was suppressed in mice infected with Δ*mqo* parasites. This suggests an essential role for MQO in the fumarate cycle. OAA produced by MQO in mitochondria is transported to the cytosol by a dicarboxylate–tricarboxylate carrier homolog (DTC) [25] and converted to aspartate, which is necessary for the purine salvage pathway (see Additional file 1) [11].

The viability and growth of *P. berghei* was reduced by deficiency of MQO. MQO reduces UQ to UQH_2_, which is associated with chemiosmotic gradient maintenance and ATP synthesis via ETC and oxidative phosphorylation in the mitochondria [11, 12]. Previous studies have suggested that the gene encoding the β subunit of mitochondrial ATP synthase (mATPβ) could be disrupted in *P. berghei* [26]. The growth of asexual *P. berghei* parasites was delayed by deficiency of mATPβ; however, it is unknown whether mice infected with mATPβ-deficient *P. berghei* develop ECM [26]. These findings suggest that MQO-mediated mitochondrial function is required for full virulence of asexual-blood-stage *Plasmodium* parasites.

In addition, the viability of Δ*fh* parasites was comparable to that of control parasites. Detection of host-derived FH activity suggests that host-derived FH functions in erythrocytes infected with Δ*fh* parasites are involved in maintain fumarate metabolism. FH is localized in the mitochondria and cytosol in all eukaryotes [27, 28]. In Δ*fh* parasites, fumarate, which is generated via the purine nucleotide cycle, may be secreted into reticulocytes and converted to malate by host-derived FH; malate ultimately enters the fumarate cycle via MQO in malaria parasites.

In this study, fumarate or malate supplementation increased the luciferase activity of FH- and MQO-deficient parasites, as well as of control parasites. These results suggest that the increase in viability is independent of malarial FH and MQO. In uninfected erythrocytes, FH and cytosolic malate dehydrogenase are present, which represents an alternative OAA biosynthesis pathway. This increased aspartate production, via AAT and pyrimidine de novo biosynthesis, enhances the growth of *Plasmodium* parasites. This hypothesis is supported by the fact that establishment of AAT-deficient malaria parasites has been unsuccessful [14].

This study suggests that FH and MQO are not essential for survival of rodent malaria parasites that mainly infects reticulocytes; indeed, cultures of FH- and MQO-deficient *P. falciparum* could not be established [9]. Srivastava et al. [14] reported that the metabolic profile in reticulocytes is enriched compared with that in mature human and mouse erythrocytes. Therefore, the interaction between host and parasite metabolism is enhanced in reticulocytes compared to in mature erythrocytes.

MQO is conserved among all apicomplexan parasites, including *Cryptosporidium* and *Perkinsus*, which are early branching groups of chromalveolates (apicomplexa and dinoflagellates, respectively). Despite the absence of genes encoding TCA cycle enzymes in the genome of *Cryptosporidium parvum*, few mitochondrial enzymes—such as MQO, type-II NADH dehydrogenase and cyanide-insensitive alternative oxidase—are conserved in the *C. parvum* mitosome [29]. This observation led to the hypothesis that MQO plays a structural rather than metabolic role in these parasites [9]. The inner membrane structure of mouse mitochondria is regulated by MMP [30]. The effect of MQO on MMP and inner membrane structure was evaluated using MitoTracker^®^ Red CMXRos and BODIPY FL C16, respectively. However, no obvious change in MMP or inner membrane structure was detected in Δ*mqo* parasites, indicating that the primary role of MQO is metabolic rather than structural, at least in *P. berghei* parasites.

## Conclusion

Anti-malarial atovaquone kills both the blood and liver stages of *Plasmodium* parasites [31], and atovaquone-resistant parasites cannot be transmitted to other hosts [32]; this indicates that mitochondrial function is necessary for the viability and growth of *Plasmodium* parasites at all lifecycle stages. In *P. falciparum*, a functional ETC is important for survival of blood-stage parasites [31], while both the TCA cycle and the ETC are critical for parasite development at the insect stage [7]. In *Plasmodium* parasites, the enzymes for pyrimidine salvage and purine de novo pathways are not conserved; therefore, these parasites rely solely on the pyrimidine de novo and purine salvage pathways for pyrimidine and purine production. MQO is a key enzyme in supplying OAA for the fumarate cycle, which produces aspartate in the cytosol to feed the pyrimidine de novo and purine salvage pathways [9, 13]. Hence, MQO is a trifunctional enzyme involved in the TCA cycle, ETC and fumarate cycle. The finding that MQO, but not FH, is involved in both the viability and growth of asexual-blood-stage parasites and development of ECM suggest it to be a potential target for the treatment and prevention of the transmission of severe malaria.

## Additional files

**Additional file 1.** Fumarate cycle in *Plasmodium falciparum*. Fumarate, which is generated via purine biosynthesis, is converted into malate by fumarate hydratase (FH) [11]. Then, malate is converted to oxaloacetate (OOA) by malate:quinone oxidoreductase (MQO) [12]; the oxidation of malate to OOA generates ubiquinol (UQH_2_), which feeds the electron transport chain [11, 12]. Two of the eight mitochondrial TCA cycle enzymes, FH and MQO, may be essential for survival of asexual-blood-stage *P. falciparum* [9]. Note: MQO is conserved among all apicomplexan parasites, including all *Cryptosporidium* species [29].

**Additional file 2.** The sequence of primer used in this study.

**Additional file 3.** Generation of FH- and MQO-deficient *Plasmodium berghei*. SK-1 vector (A) and Sk-1-luc2 vector (B). Restriction sites of NheI and BglII restriction enzymes were shown. Schematic representation of gene-targeting vectors (A and B). Luciferase (luc2)-expressing cassette was introduced into target gene by double-crossover homologous recombination. Arrows (F1, F2, M1 and M2) denote primers specific for the 5′ and 3′ regions of the target gene (see Additional file 2). (A) Introduction of luc2-expressing cassette into the *fh* locus (PBANKA_082810) of *P. berghei* parasites. Proper integration was confirmed using primers specific for *fh* (WT, 3.0 kbp; Δ*fh*, 6.8 kbp) for three cloned transfected parasites. (B) Introduction of luc2-expressing cassette into the *mqo* locus (PBANKA_111630) of *P. berghei* parasites. Proper integration was confirmed using primers specific for *mqo* (WT, 3.0 kbp; Δ*mqo*, 7.0 kbp) for three cloned transfected parasites.

**Additional file 4.** Deficiency of FH and MQO has no effect on gametocyte production. Blood was obtained from infected mice showing 3% parasitaemia and cultured for 22 h under standardized *in vitro* culture conditions. Then, mature gametocytes and schizonts were collected by Nycodenz density-gradient centrifugation. (A and B) Expression of gametocyte-specific genes. *mdv-1/peg3* [21] and *g377* [22] were subjected to semi-quantitative RT–PCR using specific primers (see Additional files 2, 3). The *hsp70* was used as a positive control. Samples treated with DNase-treated RNA template (*hsp70* (-)) were used as a negative control that is the control of eventual DNA contamination of the RNA preparations. Experiments were performed in duplicate and representative data are shown. (C) Control and Δ*fh* parasites-infected erythrocytes cultured for 22 h. (D) Control and Δ*mqo* parasites-infected erythrocytes cultured for 22 h. White arrows indicate representative mature gametocytes. The scale bars indicate 20 μm. Note that sex-specific features such as nuclear enlargement, the distribution of pigment granules throughout the cytoplasm and enlargement of the cells are observed in both Δ*fh*- and Δ*mqo*-parasite cultures just the same as reported by Mons [23].

**Additional file 5.** Parasitaemia is suppressed by deficiency of parasite MQO but not FH. Bar graph of parasitaemia in Fig. 5A. Parasitaemia on days 3–7 post-infection were shown. Asterisks indicate a statistically significant difference (*, *vs.* control).

## Abbreviations

DCIP: 2,6-dichlorophenolindophenol
APAD^+^: 3-acetylpyridine-adenine dinucleotide, oxidized form
ATP: adenosine triphosphate
AAT: aspartate aminotransferase
DTC: dicarboxylate–tricarboxylate carrier homolog
ETC: electron transport chain
ECM: experimental cerebral malaria
FH: fumarate hydratase
*hdhfr*: human dihydrofolate reductase-thymidylate synthase
MQO: malate:quinone oxidoreductase
NBT: nitroblue tetrazolium
OAA: oxaloacetate
PEP: phosphoenolpyruvate
PEPC: phosphoenolpyruvate carboxylase
TCA: tricarboxylic acid
UQH_2_: ubiquinol
UQ: ubiquinone
mATPβ: β subunit gene of mitochondrial ATP synthase
