# Supplementary material for: Suppression of experimental cerebral malaria by disruption of malate:quinone oxidoreductase
Source: Malar J. 2017 Jun 12;16:247. doi: 10.1186/s12936-017-1898-5 (PMC5469008; doi:10.1186/s12936-017-1898-5)
Supplement: Supplementary file 1 — Additional file 1. Fumarate cycle in Plasmodium falciparum. Fumarate, which is generated via purine biosynthesis, is converted into malate by fumarate hydratase (FH) [11]. Then, malate is converted to oxaloacetate (OOA) by malate:quinone oxidoreductase (MQO) [12]; the oxidation of malate to OOA generates ubiquinol (UQH2), which feeds the electron transport chain [11, 12]. Two of the eight mitochondrial TCA cycle enzymes, FH and MQO, may be essential for survival of asexual-blood-stage P. falciparum [9]. Note: MQO is conserved among all apicomplexan parasites, including all Cryptosporidium species [29]. [file 12936_2017_1898_MOESM1_ESM.doc]

**Additional files**

**
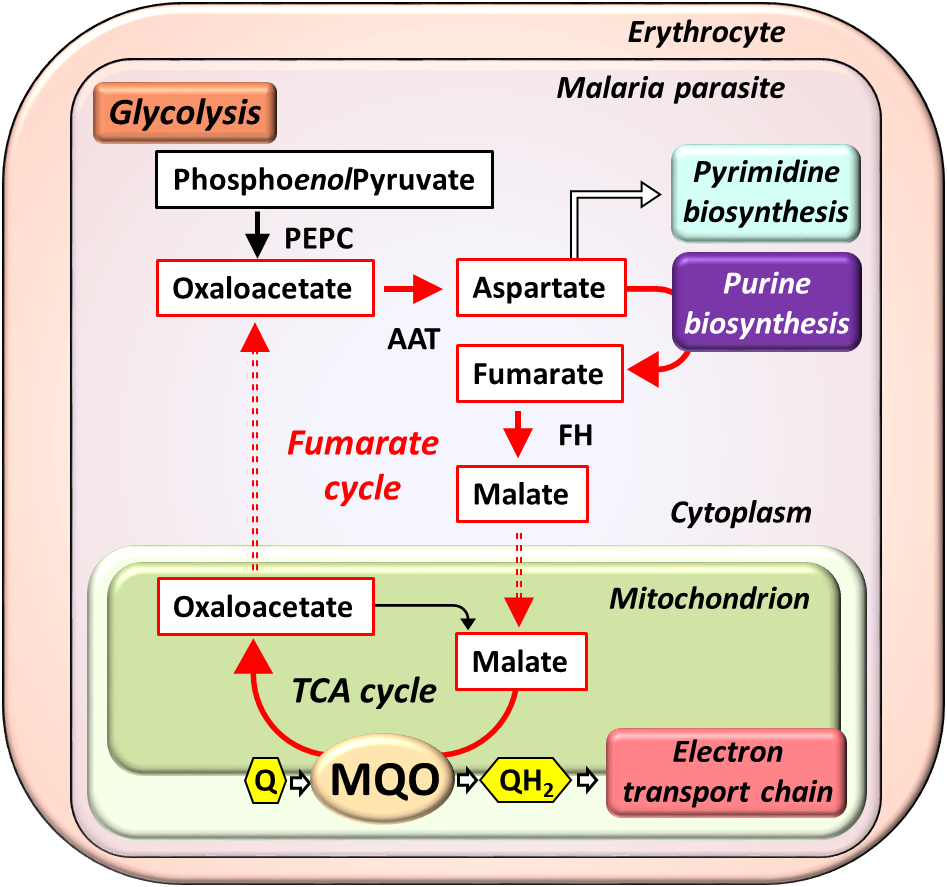
**

**Additional file 1. Fumarate cycle in *Plasmodium falciparum***

Fumarate, which is generated via purine biosynthesis, is converted into malate by fumarate hydratase (FH) [11]. Then, malate is converted to oxaloacetate (OOA) by malate:quinone oxidoreductase (MQO) [12]; the oxidation of malate to OOA generates ubiquinol (UQH_2_), which feeds the electron transport chain [11, 12]. Two of the eight mitochondrial TCA cycle enzymes, FH and MQO, may be essential for survival of asexual-blood-stage *P. falciparum* [9]. Note: MQO is conserved among all apicomplexan parasites, including all *Cryptosporidium* species [29].
