# Supplementary material for: Suppression of experimental cerebral malaria by disruption of malate:quinone oxidoreductase
Source: Malar J. 2017 Jun 12;16:247. doi: 10.1186/s12936-017-1898-5 (PMC5469008; doi:10.1186/s12936-017-1898-5)
Supplement: Supplementary file 2 — Additional file 2. The sequence of primer used in this study. [file 12936_2017_1898_MOESM2_ESM.doc]

**Additional file 2. The sequence of primer used in this study.**
