# Supplementary material for: Suppression of experimental cerebral malaria by disruption of malate:quinone oxidoreductase
Source: Malar J. 2017 Jun 12;16:247. doi: 10.1186/s12936-017-1898-5 (PMC5469008; doi:10.1186/s12936-017-1898-5)
Supplement: Supplementary file 3 — Additional file 3. Generation of FH- and MQO-deficient Plasmodium berghei. SK-1 vector (A) and Sk-1-luc2 vector (B). Restriction sites of NheI and BglII restriction enzymes were shown. Schematic representation of gene-targeting vectors (A and B). Luciferase (luc2)-expressing cassette was introduced into target gene by double-crossover homologous recombination. Arrows (F1, F2, M1 and M2) denote primers specific for the 5′ and 3′ regions of the target gene (see Additional file 2). (A) Introduction of luc2-expressing cassette into the fh locus (PBANKA_082810) of P. berghei parasites. Proper integration was confirmed using primers specific for fh (WT, 3.0 kbp; Δfh, 6.8 kbp) for three cloned transfected parasites. (B) Introduction of luc2-expressing cassette into the mqo locus (PBANKA_111630) of P. berghei parasites. Proper integration was confirmed using primers specific for mqo (WT, 3.0 kbp; Δmqo, 7.0 kbp) for three cloned transfected parasites. [file 12936_2017_1898_MOESM3_ESM.doc]

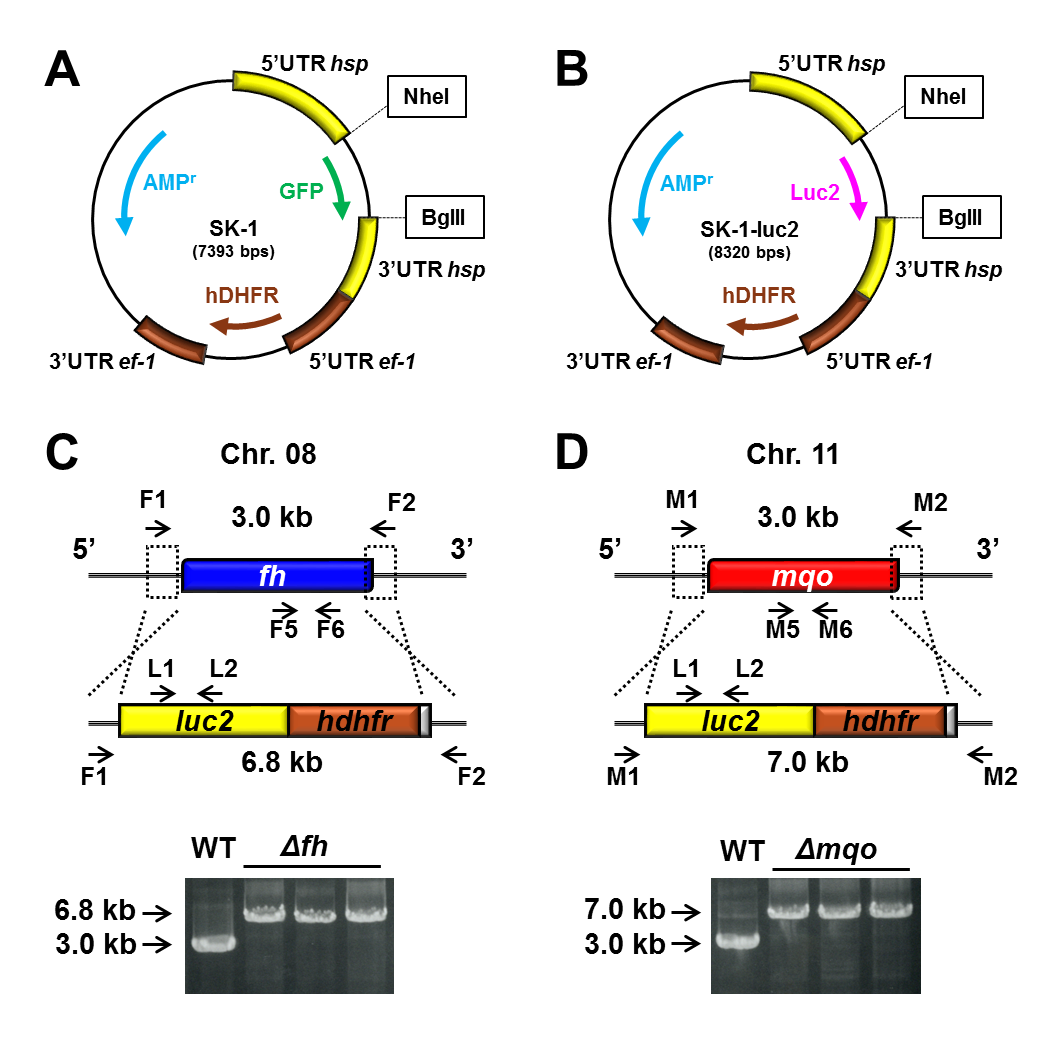


**Additional file 3** G**eneration of FH- and MQO-deficient *Plasmodium berghei***

SK-1 vector (A) and Sk-1-luc2 vector (B). Restriction sites of NheI and BglII restriction enzymes were shown. Schematic representation of gene-targeting vectors (A and B). Luciferase (luc2)-expressing cassette was introduced into target gene by double-crossover homologous recombination. Arrows (F1, F2, M1 and M2) denote primers specific for the 5′ and 3′ regions of the target gene (see Additional file 2). (A) Introduction of luc2-expressing cassette into the *fh* locus (PBANKA_082810) of *P. berghei* parasites. Proper integration was confirmed using primers specific for *fh* (WT, 3.0 kbp; *Δfh*, 6.8 kbp) for three cloned transfected parasites. (B) Introduction of luc2-expressing cassette into the *mqo* locus (PBANKA_111630) of *P. berghei* parasites. Proper integration was confirmed using primers specific for *mqo* (WT, 3.0 kbp; *Δmqo*, 7.0 kbp) for three cloned transfected parasites.
