# Supplementary material for: Suppression of experimental cerebral malaria by disruption of malate:quinone oxidoreductase
Source: Malar J. 2017 Jun 12;16:247. doi: 10.1186/s12936-017-1898-5 (PMC5469008; doi:10.1186/s12936-017-1898-5)
Supplement: Supplementary file 4 — Additional file 4. Deficiency of FH and MQO has no effect on gametocyte production. Blood was obtained from infected mice showing 3% parasitaemia and cultured for 22 h under standardized in vitro culture conditions. Then, mature gametocytes and schizonts were collected by Nycodenz density-gradient centrifugation. (A and B) Expression of gametocyte-specific genes. mdv-1/peg3 [21] and g377 [22] were subjected to semi-quantitative RT–PCR using specific primers (see Additional files 2, 3). The hsp70 was used as a positive control. Samples treated with DNase-treated RNA template (hsp70 (-)) were used as a negative control that is the control of eventual DNA contamination of the RNA preparations. Experiments were performed in duplicate and representative data are shown. (C) Control and Δfh parasites-infected erythrocytes cultured for 22 h. (D) Control and Δmqo parasites-infected erythrocytes cultured for 22 h. White arrows indicate representative mature gametocytes. The scale bars indicate 20 μm. Note that sex-specific features such as nuclear enlargement, the distribution of pigment granules throughout the cytoplasm and enlargement of the cells are observed in both Δfh- and Δmqo-parasite cultures just the same as reported by Mons [23]. [file 12936_2017_1898_MOESM4_ESM.doc]

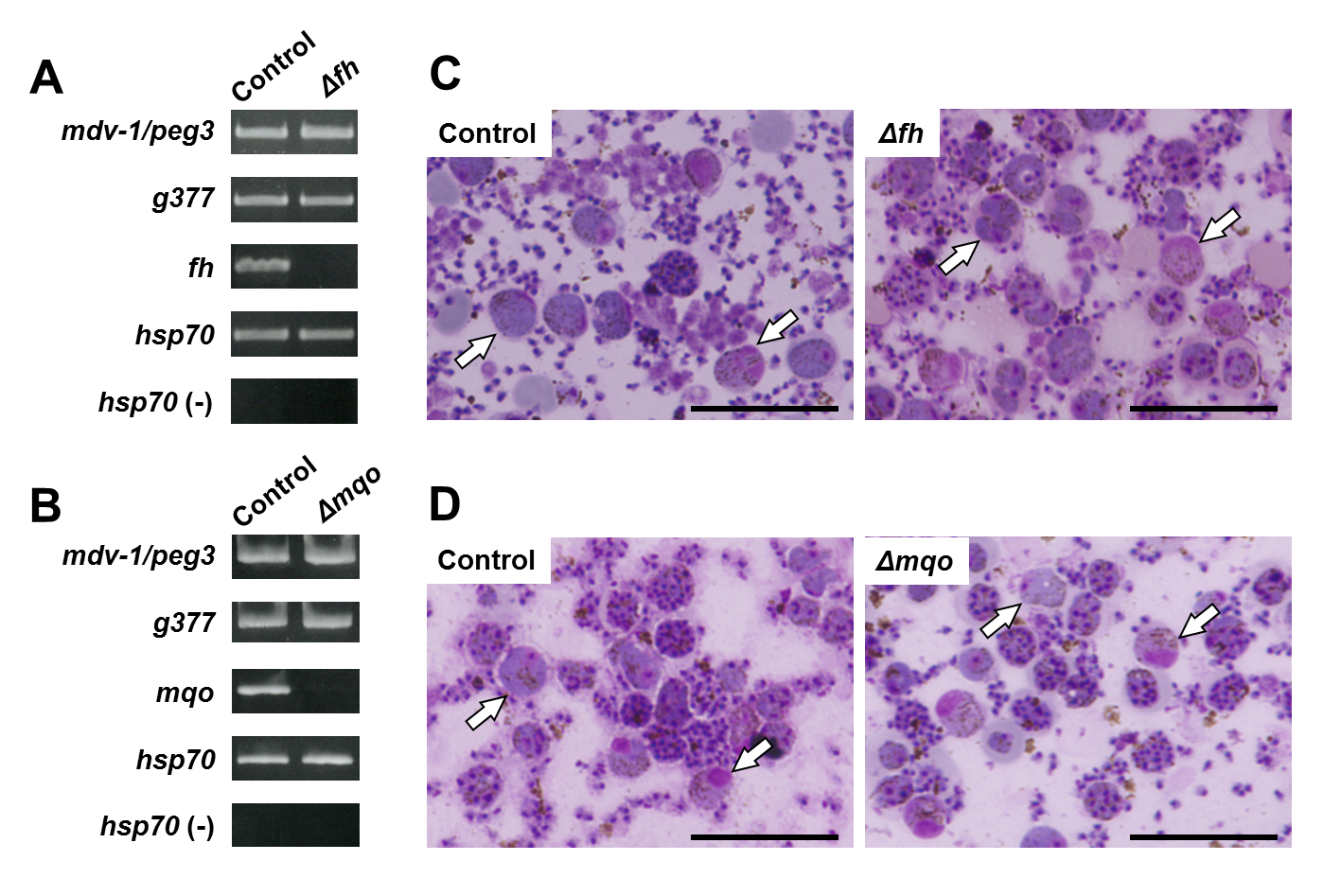


**Additional file 4. Deficiency of FH and MQO has no effect on gametocyte production.** Blood was obtained from infected mice showing 3% parasitaemia and cultured for 22 h under standardized *in vitro* culture conditions. Then, mature gametocytes and schizonts were collected by Nycodenz density-gradient centrifugation. (A and B) Expression of gametocyte-specific genes. *mdv-1/peg3* [21] and *g377* [22] were subjected to semi-quantitative RT–PCR using specific primers (see Additional file 2 and 3). The *hsp70* was used as a positive control. Samples treated with DNase-treated RNA template (*hsp70* (-)) were used as a negative control that is the control of eventual DNA contamination of the RNA preparations. Experiments were performed in duplicate and representative data are shown. (C) Control and *Δfh* parasites-infected erythrocytes cultured for 22 h. (D) Control and *Δmqo* parasites-infected erythrocytes cultured for 22 h. White arrows indicate representative mature gametocytes. The scale bars indicate 20 μm. Note that sex-specific features such as nuclear enlargement, the distribution of pigment granules throughout the cytoplasm and enlargement of the cells are observed in both *Δfh*- and *Δmqo*-parasite cultures just the same as reported by Mons [23].
