# Supplementary material for: Suppression of experimental cerebral malaria by disruption of malate:quinone oxidoreductase
Source: Malar J. 2017 Jun 12;16:247. doi: 10.1186/s12936-017-1898-5 (PMC5469008; doi:10.1186/s12936-017-1898-5)
Supplement: Supplementary file 5 — Additional file 5. Parasitaemia is suppressed by deficiency of parasite MQO but not FH. Bar graph of parasitaemia in Fig. 5A. Parasitaemia on days 3–7 post-infection were shown. Asterisks indicate a statistically significant difference (*, vs. control). [file 12936_2017_1898_MOESM5_ESM.doc]

**
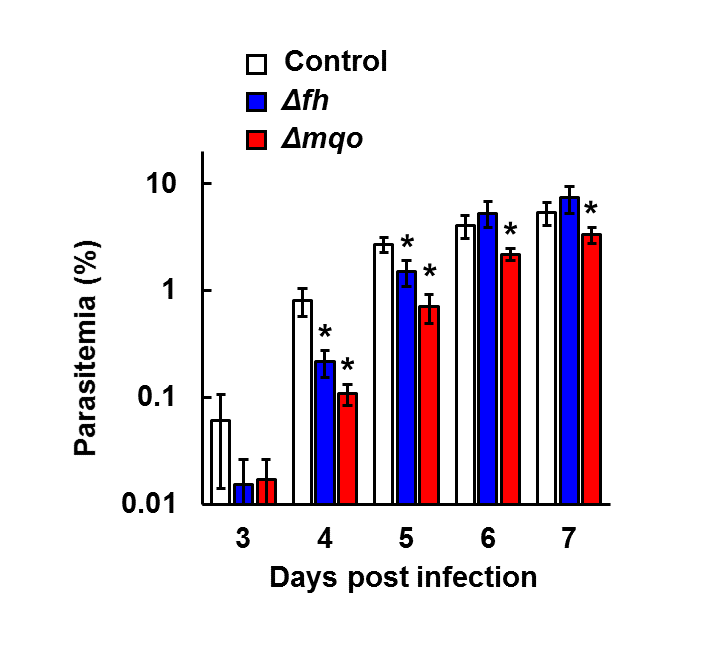
**

**Additional file 5. Parasitaemia is suppressed by deficiency of parasite MQO but not FH.** Bar graph of parasitaemia in Fig. 5A. Parasitaemia on days 3-7 post-infection were shown. Asterisks indicate a statistically significant difference (*,*vs.* control).
